# Genesee County Medical Society—Semi-Annual Meeting

**Published:** 1868-06

**Authors:** 


### Genesee County Medical Society—Semi-Annual Meeting.

Batavia, June 9th, 1868.

The semi-annual meeting of the Genesee County Medical Society was held at Batavia, June 9th, 1868. Present—O. R. Croff, President. in the chair, L. B. Cotes, J. R. Cotes, M. W. Townsend, A. P. Jackson, E. B. Lounsbury, G. W. Croff, L. L. Tozier, H. D. Benham, J. C. Watson, J. Root, J. S. Billings, A. G. Ellenwood and M. C. Potter.

The following communication was received:

To the Secretary of the Genesee County Medical Society:

Sir:—The undersigned, a member of the Genesee County Medical Society, has to respectfully report, that a fellow member of this Society has been guilty of a violation of the code of ethics of the National Medical Association and of the By-Laws of this Society in the following manner, to-wit: Dr. N. G. Clark did in the month of April or May, 1868, consult with a homœopathic practitioner by the name of Hutchins, in the family of Wm. Terry of Batavia. The undersigned has the honor to submit this case for the action of the Society, praying that the code under which we live may not be violated without just notice.

Your obedient servant,

M. W. Townsend.

On motion, the subject matter of the above communication was referred to a committee of three, M. W. Townsend, J. Root and M. C. Potter, who made the following report:

The committee appointed to report on the charges preferred against Dr. N. G. Clark, have the honor to submit the following:

*Whereas*, Dr. Clark has been guilty of gross violation of the Code of Ethics and By-Laws of this Society in consulting with an irregular practitioner in the manner and form mentioned in the charges;

*Resolved*, That Dr. N. G. Clark ought to be, and hereby is, expelled from this Society, and that the Secretary be instructed to give him official notice.

Respectfully,

M. W. Townsend,
J. Root,
M. C. Potter,
Committee.

The report was accepted and the resolution *passed unanimously.*

Dr. Townsend also moved that hereafter it shall be considered dishonorable for any member of the regular profession to hold medical consultation with Dr. N. G. Clark, and that the proceedings of this meeting, signed by the President and Secretary, be published in the Buffalo Medical Journal. Passed unanimously.

L. L. Tozier, Sec'y. O. R. Croff, President.

